# Supplementary material for: Comparative analysis of pulmonary ventilation distribution between low-cost and branded incentive spirometers using electrical impedance tomography in healthy adults: Study protocol
Source: PLoS One. 2024 May 6;19(5):e0302476. doi: 10.1371/journal.pone.0302476 (PMC11073695; doi:10.1371/journal.pone.0302476)
Supplement: S2 File — (PDF) [file pone.0302476.s002.pdf]

METHODS

Study Design:

Crossover clinical trial, in which the distribution of pulmonary ventilation will be evaluated by means of electrical impedance tomography, during the use of a commercial flow respiratory incentive and the modified Pachón incentive, in a sample of healthy people from the city of Santiago from cali. Both incentives will be used in all participants, and the order of assignment will be randomized.

Sample:

Sample size of 30 subjects (15 men, 15 women) healthy volunteers from the Municipality of Santiago de Cali. The type of sampling is non-probabilistic for convenience.

Distribution Age Ranges:

Age Ranges	Men	Women
18 – 34	5	5
35 – 50	5	5
51 – 65	5	5

Selection criteria

- **Inclusion criteria**:
  - People aged between 18-65 years
  - People with clinical stability, defined as the absence of any acute illness during the previous 6 weeks and a Charlson index score of 0-1
  - People with body mass index (BMI) 18.5 – 35 Kg/m2 (60.61)
  - People without mental or cognitive disorders
  - People who accept informed consent

- **Exclusion criteria:**
  - People with pacemakers, cardioverters or cardio defibrillators.
  - People with metal implants
  - People with any condition in which the CT signal registration is low
  - Pregnant women
  - Participants with injuries, skin changes or presence of devices that prevent placement of the electrode belt around the chest.

  - People with a high level of physical activity according to the IPAQ short version questionnaire
  - People whose spirometry records obstruction or restriction
  - People who do not understand the verbal command of the incentive technique}

- **Dropout criteria**
  - Request by the subject to be excluded from the protocol expressed verbally or in writing

Study Variables

- **Primary variables:**

  - **Delta EELI (ΔEELI):** It shows changes in end-expiratory lung impedance before and after an intervention, which can be interpreted as end-expiratory lung volume changes in the plane where the electrodes are located. It represents the functional residual capacity, and at the same time is directly related to lung recruitment
  - **Tidal Variation (VT):** Sum of the regional relative impedance changes of the entire state image or within the defined region of interest (ROI). It is related to the increase in resistance of the lung tissue to the passage of current, which increases with the entry of air and decreases with the exit of this
  - **Minute variation (MV):** Global or regional minute tidal variation: average of the sum of the regional relative impedance changes in the last minute of the entire state image or within the defined region of interest.

- **Secondary variables:**

  - **IPAQ:** Instrument that provides information on estimated energy expenditure in 24 hours in different areas of daily life.
  - **Charlson Index:** Relates long-term mortality to participant comorbidity
  - **%FEV1 Pred:** Percentage of predicted forced expiratory volume in the first second
  - **%FVC Pred:** Percentage of predicted forced vital capacity

- **%FVC/FEV1:** Percentage of the predicted relationship between forced vital capacity and FEV1

Materials and Instruments:

The instruments that will be used in the study are: stadiometer, scale, spirometer, Modified Pachón Incentive, Hudson flow respiratory incentive, equipment to measure impedance (Pulmovista), data collection format. And they are described below:

- **Modified Pachón Incentive:**

Handmade device that imitates conventional respiratory incentives, but is made with recyclable, low-cost materials and is also easy to manufacture. Modified in 2004 in a research work by students from the Universidad del Valle where they analyzed the characteristics of the materials required for its design and laboratory tests were carried out, they defined that modified Pachón Incentive allows mobilize flows that range from 600 cc/sec to more than 1400 cc/sec, which characterizes it as a flow incentive. The materials used for its construction are a buretrol and an unlubricated Today brand condom, since other condoms that have lubricant are uncomfortable to handle, cause the condom to adhere to the walls of the buretrol, and hinder its movement.

- **Triflo® II Respiratory Incentive:**

Method of promoting deep voluntary breathing, which provides participants with visual feedback of the inspiratory volume, using 3 color-coded balls in three chambers, with mouthpiece and tube. The minimum flow is marked on each chamber: 600, 900 and 1200 ml/sec. It is used to help with inspiratory muscle training and prolonged maximum inspiration, deep breathing stimulates the alveoli to fully expand, it is also often used to prevent or reverse the formation of pulmonary atelectasis. It is manufactured by HUDSON RCI.

- **Pulmovista 500®:**

It is an electrical impedance tomograph, from Dräger. Data is continuously represented in the form of images, curves and parameters to observe ventilation continuously and directly in various lung regions, as well as the changes that occur in lung volumes at the end of expiration, non-invasively, in time. really and directly next to the bed.

- **Spirometer:**

It is a device that is commonly used to evaluate how the lungs are working by measuring how much air you inhale, how much you exhale, and how quickly you exhale. Spirometry is used to diagnose asthma, chronic obstructive pulmonary disease (COPD), and other diseases that affect breathing. Spirometry can also be used periodically to monitor the condition of your lungs and check whether treatment for chronic lung disease helps you breathe better. For this study, a Medgraphics Cardiorespiratory Diagnostics® spirometer was used, with the variables analyzed through Breeze Suite 6.4.1.44 SP4 software.

- **Stadiometer:**

It is a height meter that is fixed to the wall or a support and is used to accurately measure people, so that when placed under it, the stadiometer will rest on your head indicating on the dial the exact height of who you are using it. For this study, a SECA model 213® stadiometer was used.

- **Weighing machine:**

Device used for measuring weights. For this study, a SECA® brand scale was used.

- **Data collection format**

For this research, a data collection format was designed (Annex 3), a format created by the researcher composed of a section of sociodemographic information (age, date of birth, gender, sociodemographic stratum, occupation, education and marital status), background personal and family (the Charlson index will be applied to evaluate associated comorbidities, anthropometric data (weight and height), physical activity level (IPAQ), lung function data (spirometry) and results on ventilation and global and regional lung impedance of each individual.

To measure clinical stability and the level of physical activity, the following instruments were used:

- **Charlson index:**

It is a system for evaluating life expectancy, depending on the age at which it is evaluated, and the comorbidities of the subject. In addition to age, it consists of 19 pathology items with their respective qualification (Annex 4), which, if present, have been proven to have a specific influence on the subject's life expectancy. In general, absence of comorbidity is considered: 0-1 points, low comorbidity: 2 points and high comorbidity > 3 points. Prediction of mortality less than 3 years: score of 0: (12% mortality/year); from 1-2: (26%mortality/year); from 3-4: (52% mortality/year); and > 5: (85% mortality/year).

- **International Physical Activity Questionnaire (IPAQ) short versión:**

IPAQ researchers developed several versions of the instrument according to the number of questions (short or long). The short version consists of 7 items and provides information about the time the person spends doing moderate and vigorous intensity activities, walking, and sitting. Especially recommended when population monitoring is intended in research.

Classification of physical activity levels according to the criteria established by the IPAQ

High physical activity level	Report 7 days a week of any combination of walking, or moderate or high intensity activities achieving a minimum of 3,000 MET-min/week;<br>when vigorous activity is reported at least 3 days a week reaching at least 1,500 MET-min/week
Moderate physical activity level	Report 3 or more days of vigorous activity for at least 20 minutes daily;<br>When 5 or more days of moderate activity and/or walking for at least 30 minutes a day are reported;<br>When describing 5 or more days of any combination of walking and moderate or vigorous activities achieving at least 600 MET-min/week
Low physical activity level	It is defined when the subject's level of physical activity is not included in the high or moderate categories.

Procedures:

- **Phase 1: Preparation for the study**

At this stage, the search for initial information will be carried out, for this a bibliographic review will be carried out for the present study that will include research published in different databases, related to the validation and comparison of medical devices, the use of electrical impedance tomography. and studies related to the use

of respiratory incentive in the clinical setting and their levels of evidence. Once this is done, the research approach, the study design, the way of selection and sample size, and the writing of the document will be carried out. To carry out this study, the main researcher will carry out a 24-hour training in the use of the equipment with the staff of the commercial house. (Dräger). The informed consent will be designed and the written research work will be sent to the ethics committee to be evaluated and receive endorsement.

- **Phase 2: Instrument Design and Adjustment**

The data collection instrument will be designed.

For the standardization of anthropometric measurements (weight and height), standardized operating procedures will be used and will be carried out according to the instructions defined in these

For the measurement with the electrical impedance tomograph, the standardized operating procedure designed for projects previously carried out by the Cardiopulmonary Health and Exercise Research Group will be used, and it will be carried out according to the instructions defined therein.

A series of intra-observer tests will be carried out for the use of the PulmoVista 500 in order to increase the reliability of the variables to be measured with this equipment.

The pilot test will be carried out with 4 healthy people who were not part of the sample, they will be informed about the research, they will be asked to sign the informed consent and the measurements and data collection will continue with the data collection forms. data designed by the researcher. This pilot test will be used to make adjustments to the protocol and data collection formats based on the information collected; The duration, execution and questions generated during the process will be recorded.

- **Phase 3: Data Collection:**

    - **Call for participants**

To recruit healthy volunteers between 18-65 years of age from the community of Santiago de Cali, direct contact will be made with people from the administrative areas of the Universidad del Valle, family and friends to whom information about the objectives will initially be provided. of the research and will be invited to participate.

Social networks will also be used to send research information and the contacts (email, cell phone) of the researchers will be left so that participants can clarify any questions.

People who agree to participate in the study will be contacted and the date, time and place will be agreed upon, taking into account time availability. The electrical impedance tomography will be performed in the movement laboratory located at the Universidad del Valle and the spirometries at the Hospital Universitario del Valle.

- **Data collection protocol**

Before performing spirometry

If the subject is eligible, a series of recommendations will be made that must be taken into account prior to taking measurements:

1. Not having done physical activity in the last 12 hours prior to the exam.
2. Not having smoked a cigarette in the last 12 hours prior to the exam.
3. Do not consume caffeinated drinks on the day of the exam
4. Comfortable clothes

- **Performing spirometry**

This measurement will be carried out at the Hospital Universitario del Valle, in the physical medicine and rehabilitation unit, in the pulmonary function laboratory, with the medgraphics cardiorespiratory diagnostics® brand spirometer that belongs to the Universidad del Valle and is in custody. from the University Hospital. The spirometries will be carried out by the researcher, who is a physiotherapist specializing in cardiopulmonary physiotherapy. The day of the test will begin with the completion of the informed consent, this will be read and explained to the participant and will continue with the signing. On this day, the researcher will fill out the participant's sociodemographic data, weight and height measurement, Charlson index and abbreviated IPAQ questionnaire, according to the instructions defined in the standardized operating procedures for the application of these procedures in the data collection format.

Spirometry will be performed according to the institutional protocol of the Hospital Universitario del Valle. The duration of this procedure will take approximately 30 minutes

- **Randomization**

To assign the first respiratory incentive with which each participant will begin the first measurement, simple randomization will be used through the Randomization.com website ⟨http://www.randomization.com⟩.

- **Measurement of ventilation with electrical impedance tomography in lung reexpansion training with the modified Pachón Incentive and with the commercial respiratory incentive**

These measurements will be carried out at the facilities of the Cardiopulmonary Health and Exercise Research Group, according to the availability of spaces and equipment. The first measurement will be carried out with the incentive that has been randomly assigned to the participant, the second measurement will be carried out a week later with the other incentive, with the objective of completing the washout period (Figure X), both measurements will be carried out by the main researcher. For the measurement of electrical impedance tomography, the instructions defined in the standardized operating procedure designed for this will be followed and for the application of the protocol for the use of incentives, the instructions defined in the standardized operating procedures designed for each respiratory incentive to be used will be followed. The duration of this procedure will be approximately 30 minutes for each measurement.

Figure X: Study design diagram

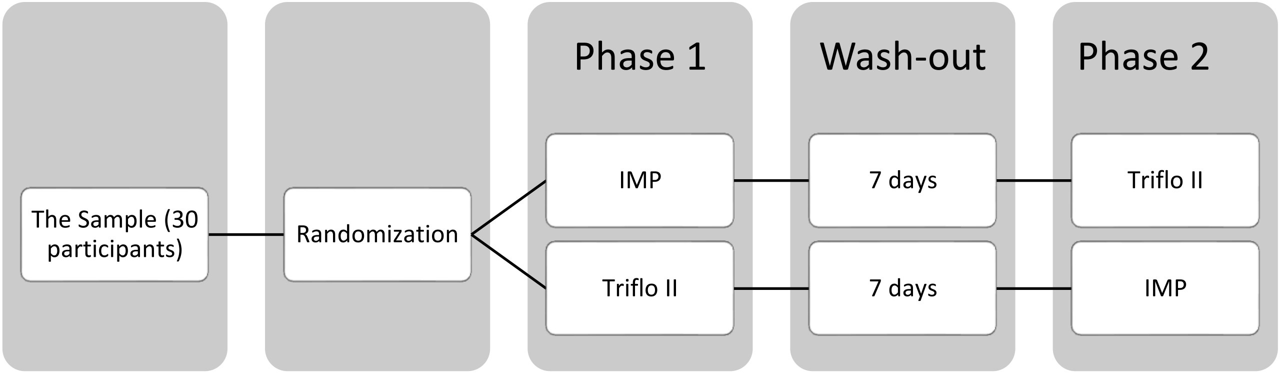

IMP (modified Pachón incentive)

- **Measurement moments of electrical impedance tomography:**

Electrical impedance tomography measurement will be performed using Dräger's PulmoVista 500. The participant will be positioned in a high-back chair that allows the belt to always rest on a surface and the team's signal to be high. A belt with 16 integrated electrodes will be placed around the patient's chest between the fifth and sixth intercostal space. When choosing the belt, the diameter of the participant's chest will be taken into account to allow for an adequate fit.

Before starting the protocol, a different researcher than the one who will perform the electrical impedance tomography measurements will explain to the participant through a video the correct use of the incentive that they must use according to the randomization. Once the participant is ready to start, the recording will begin. Initially, two minutes of calm breathing will be recorded and the first event corresponding to moment 1 (M1) will be marked. Next, the lung reexpansion protocol will be carried out with the corresponding incentive.

The frequency and duration of the exercises is still controversial, however, what is currently recommended is to perform three sets of ten repetitions with a 30 to 60 second pause between each set. The protocol of this study will be carried out with three series of ten breaths with the respiratory incentive, with a rest of 30 seconds between series, during the last repetition of the last series the second event corresponding to moment 2 (M2) will be marked, and Finally, the participant will perform two minutes of calm breathing and the third event corresponding to moment 3 (M3) will be marked. (Figure Y)

Figure: Illustration of Reexpansion Protocol and each measurement momento

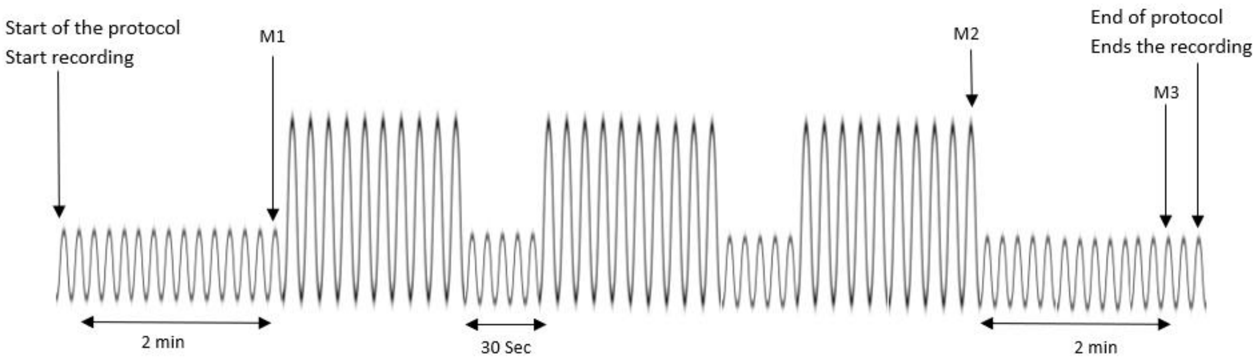

The verbal command that will be used during the use of incentives is “Take the air gently and deeply through your mouth, hold for 5 seconds, and release it gently through your mouth without removing the mouthpiece of the incentive without making any effort.” The participant will receive visual feedback from the device when the condom is raised within the buretrol in the case of the modified Pachón incentive or the spheres are raised in the Triflo II incentive.

At the beginning, during and at the end of the re-expansion maneuver, physiological variables (heart rate, respiratory rate and oxygen saturation) will be measured and 15 minutes after the end of the test to corroborate the clinical status of the participant.

To measure these vital signs, the instructions defined in the standardized operating procedures designed for this were followed.

Considering that the subjects are using a device that is not used routinely in daily life, The satisfaction of the participants after its use will be evaluated, but only for the modified Pachón incentive, which is the "new" device or the one we are testing, through a survey with a Likert rating scale with questions related to design, materials, colors and comfort during the use of the modified Pachón incentive, in the same way, it will be assessed if the perception of dyspnea by means of the Borg scale

The session will be interrupted if the participant does not wish to continue with the lung reexpansion technique, if the participant shows intolerance to the maneuver, if the respiratory rate is greater than 25 breaths per minute and if there is use of accessory muscles.

After completing each measurement with each participant, the equipment will be disinfected in accordance with the recommendations found in the disinfection protocol.

- **Data registration:**

The electrical impedance tomography variables of events M1, M2 and M3 will be taken from the “Data Analysis” window (Figure Z) that allows the review of previously recorded data files and from the “ΔEELI trend” view (Figure A) showing changes in end-expiratory lung impedance, which can be interpreted as changes in end-expiratory lung volume at the electrode plane. The analysis will focus on the spatial distribution of ventilation.

Figure Z: Image of the “Data Analysis” window

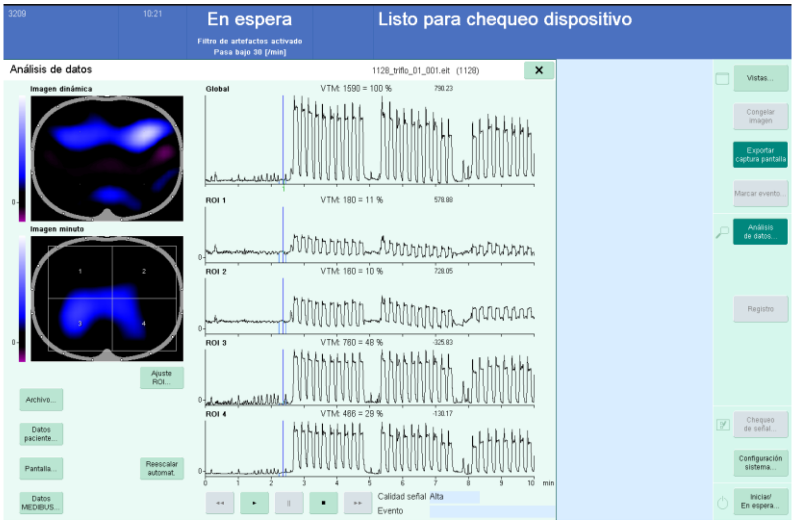

Figure A: Image of the “ΔEELI trend” window

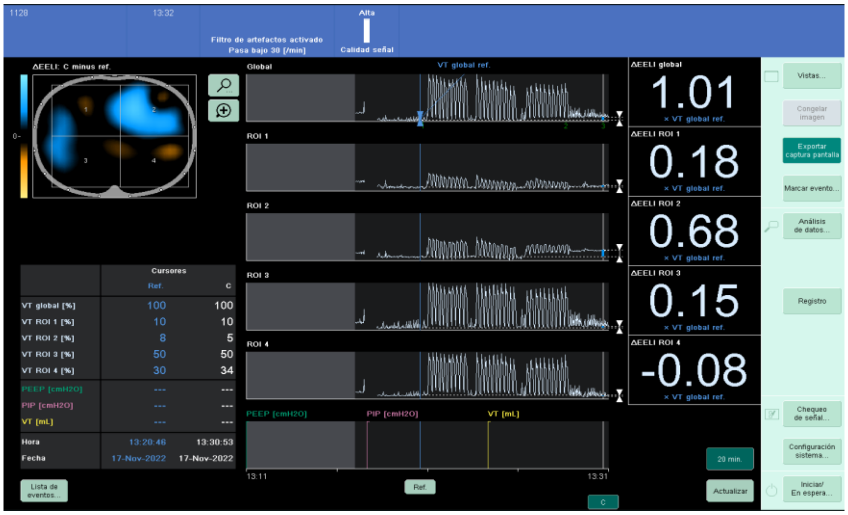

To evaluate regional ventilation, regions of interest (ROIs) will be defined (Figure B). For this purpose, the cross section of the thorax is divided into four quadrants of equal size ROI1 (right ventral), ROI2 (left ventral), ROI3 (right dorsal) and ROI4 (left dorsal). These regions of interest allow us to reflect regional changes in the distribution of lung ventilation through percentage distribution.

Figure B: regions of interest (ROIs): ROI1 (right ventral), ROI2 (left ventral), ROI3 (right dorsal) and ROI4 (left dorsal)

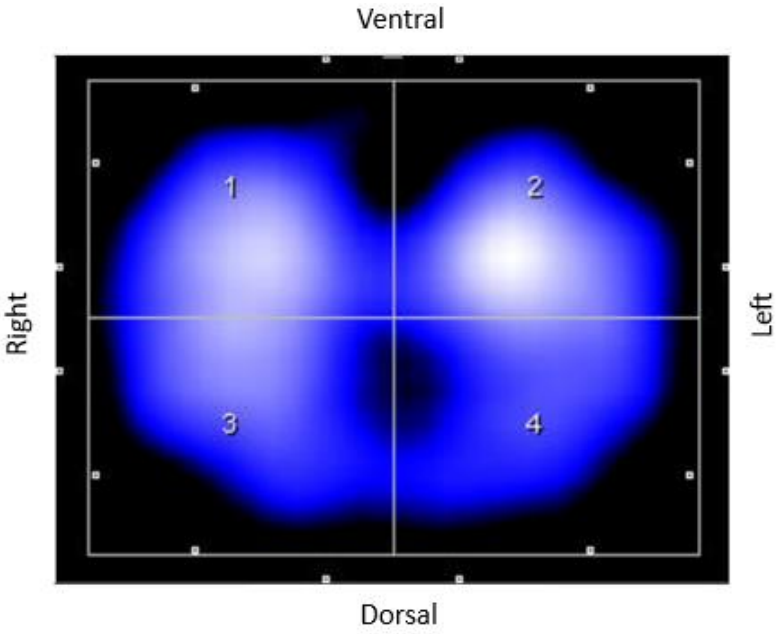

- **Quality control**

1. To guarantee the quality of the recorded information, the main researcher will carry out random reviews in which he corroborated the coherence between the original information from the tomographer, that of the registration in the forms and that of the database. Any errors detected will be corrected.
2. A person external to the study, who will not participate in the measurements, will randomly review the forms for verifying the information once every 15 days before entering it into the database. Additionally, once a month a meeting will be held with the research group in order to share progress of the project.
3. For the electrical impedance tomography data, the monitoring sessions will be recorded with the participant's code, this information will be saved in the equipment and can be consulted if necessary, a screenshot of the main measurements will be taken, These captures will be saved on a USB to be downloaded to the computer where the information collected from each participant is stored. These images will allow us to verify the information stored in the data collection format and make the corresponding corrections if necessary.
4. A checklist will be made to verify that each of the participants complies with the research step by step and that relevant information is not omitted that could generate bias or loss of information.

- **Phase 4: Statistical analysis**

Descriptive analysis

The information will be recorded in the data collection formats designed for this purpose. To then be organized in the Microsoft Excel program. In order to have an initial knowledge of the behavior of each of the variables. In the descriptive phase the data will be analyzed with IBM SPSS Statistics V23.0. Frequencies will be obtained for categorical variables, and average, median, standard deviation, quartiles, and Box plots for quantitative variables.

The Kolmogorov-Smirnov test will be used to verify the normality of the variables. For paired analyzes of variables with normal distribution, the paired t test will be used, for paired analyzes of variables with abnormal distribution, the non-parametric Wilcoxon test will be used.

For unpaired analyzes of variables with normal distribution, an unpaired T test will be used, and for unpaired analyzes with abnormal distribution, the Mann Whitney test will be used.

To analyze the differences of 3 groups onwards, the 1-way ANOVA analysis of variance with the Poshoc Dunn's test will be used. For multiple comparisons, a value of $p<0.05$ is considered for the statistical significance of the tests used.

Ethical aspects

According to the Declaration of Helsinki, the ethical principles for medical research on human beings, including the investigation of identifiable human material and information and according to the scientific and technical standards established in Resolution 8439 of 1993, for health research, and according to its Article 11. This research is classified as having minimal risk because in this project common procedures will be carried out such as anthropometric measurement of the height and weight of the subject in a healthy population, deep and forced breathing maneuvers will be requested that did not present adverse events since the participants will be healthy and these procedures did not represent physical or psychological risks. However, for the study the measurements will be carried out in a space with a flat floor, with adequate marking and accompaniment of the researchers in order to mitigate the risk of falls.

Complications from forced spirometry are rare. The most common are cough attacks, bronchospasm, chest pain, dizziness, urinary incontinence or increased intracranial pressure. Very rarely, the patient may suffer from syncopal symptoms; for this, spirometry will be performed at the Hospital Universitario del Valle, by a professional trained and experienced in performing these tests. This test will be performed to rule out the coexistence of any alteration in lung function.

In general, there are very few risks or possible complications with using the respiratory incentive, but it is important to stop if the participant feels dizzy. However, there are rare reports of pneumothorax that have been associated with the use of respiratory incentive used too aggressively in people with emphysema (and pulmonary bullae that could rupture), however these higher risks will be ruled out with the result of spirometry. in a state of normality, in addition the technique will be performed and guided by the researcher, who is a physiotherapist with experience in handling the technique and trained in case of any eventuality.

The modified Pachón incentive is a device for therapeutic use, which has industrial design registration, granted by the Superintendency of Industry and Commerce to the Universidad del Valle under the name “Inspirometer.” So its use is supported.

The Human Ethics Committee of the Universidad del Valle approved the Research protocol with approval document No. 009-022 and this research is registered in Clinicaltrials.gov (NCT05532748)

Selected subjects will sign a written informed consent form in accordance with the Declaration of Helsinki and current Good Clinical Practice guidelines. A copy of the consent will be given to each participant. The information generated by this study will be handled strictly confidentially; Privacy will be maintained and participants will not be identified in any publication.

To protect the data and security of participants, the following was considered:

The person who will carry out the measurements will undergo training with the personnel of the commercial company that distributes the Pulmovista 500 equipment to certify the qualification in carrying out the measurements.

Informed consent will be given to each of the people who will participate in the study, and each and every one of the benefits and risks to which they will be exposed will be explained.

It will be explained that the information generated is strictly confidential and will be used for academic purposes, your privacy will be maintained and you will not be identified in any publication; All data will be identified with a 4-digit sequential code, which will end with the last 2 numbers of the date on which they agreed to participate in the research.

The information will be stored digitally on the main researcher's computer, with absolute confidentiality and guaranteeing the use of a password to access it, it will be kept with strict privacy; and will be stored for 10 years, for future publications or for the development of subsequent projects if the participant authorizes it.

There is no conflict of interest in this study.
