# Supplementary material for: Comparative analysis of pulmonary ventilation distribution between low-cost and branded incentive spirometers using electrical impedance tomography in healthy adults: Study protocol
Source: PLoS One. 2024 May 6;19(5):e0302476. doi: 10.1371/journal.pone.0302476 (PMC11073695; doi:10.1371/journal.pone.0302476)
Supplement: S3 File — World Health Organization trial registration data set. (PDF) [file pone.0302476.s003.pdf]

Appendix Table

World Health Organization Trial Registration Data Set

Item	Description
1. Primary registry and trial-identifying number	The Human Ethics Committee of the Universidad del Valle approved the Research protocol with approval document No. 009-022 and assigns it the internal code: 031-022.
2. Date of registration in primary registry	May 16, 2022
3. Secondary identifying numbers	Clinicaltrials.gov (NCT05532748)
4. Sources of monetary or material support	Universidad del Valle<br>Cardiopulmonary Health and Exercise Research Group<br>own resources
5. Primary sponsor	There is no sponsor
6. Secondary sponsor(s)	There is no sponsor
7. Contact for public queries	E-mail address: nora.elena.mera@correounivalle.edu.co<br>Telephone number: +57 3187983544<br>Postal address: Carrera 12 # 20-107 house 27, Postal Code: 763533
8. Contact for scientific queries	PRINCIPAL INVESTIGATOR<br>Name: Noraelena Mera Quintero<br>Title: Physiotherapist specializing in Cardiopulmonary physiotherapy<br>e-mail address: nora.elena.mera@correounivalle.edu.co<br>Telephone number: +57 3187983544<br>Postal address: Carrera 12 # 20-107 house 27<br>Postal Code: 763533<br>Affiliation: Universidad del Valle
9. Public title	Comparative analysis of pulmonary ventilation distribution between low-cost and branded incentive spirometers using electrical impedance tomography in healthy adults.
10. Scientific title	Distribution of pulmonary ventilation with the Modified Pachón Incentive vs another commercial respiratory incentive, through electrical impedance tomography, in a healthy population of the city of Cali
11. Countries of recruitment	Colombia

12. Health condition(s) or problem(s) studied	Distribution of lung ventilation in healthy population
13. Intervention(s)	Intervention 1: The participant must use the Modified Pachón Incentive, performing 3 sets of 10 repetitions, with a 30-second rest between sets<br>Intervention 2: The participant must use the commercial Respiratory Incentive, performing 3 sets of 10 repetitions, with a 30-second rest between sets.
14. Key inclusion and exclusion criteria	Inclusion criteria:<br>• People aged between 18-65 years<br>• People with clinical stability, defined as the absence of any acute illness during the previous 6 weeks and a Charlson index score of 0-1<br>• People with body mass index (BMI) 18.5 – 35 Kg/m2 (60.61)<br>• People without mental or cognitive disorders<br>• People who accept informed consent<br><br>Exclusion criteria:<br>• People with pacemakers, cardioverters or cardio defibrillators.<br>• People with metal implants<br>• People with any condition in which the CT signal registration is low<br>• Pregnant women<br>• Participants with injuries, skin changes or presence of devices that prevent placement of the electrode belt around the chest.<br>• People with a high level of physical activity according to the IPAQ short version questionnaire<br>• People whose spirometry records obstruction or restriction<br>• People who do not understand the verbal command of the incentive technique<br><br>Dropout criteria<br>Request by the subject to be excluded from the protocol expressed verbally or in writing
15. Study type	Crossover clinical trial, in which the distribution of pulmonary ventilation will be evaluated by means of electrical impedance tomography, during the use of a commercial flow respiratory incentive and the modified Pachón incentive, in a sample of healthy people from

	the city of Santiago from cali. Both incentives will be used in all participants, and the order of assignment will be randomized.<br>**Randomization**<br>To assign the first respiratory incentive with which each participant will begin the first measurement, simple randomization will be used through the Randomization.com website ⟨http://www.randomization.com
16. Date of first enrollment	October 1, 2022
17. Target sample size	Sample size of 30 subjects (15 men, 15 women) healthy volunteers
18. Recruitment status	Complete: Participants are no longer being recruited or enrolled
19. Primary outcome(s)	**Delta EELI (ΔEELI):** It shows changes in end-expiratory lung impedance before and after an intervention, which can be interpreted as end-expiratory lung volume changes in the plane where the electrodes are located. It represents the functional residual capacity, and at the same time is directly related to lung recruitment<br>**Tidal Variation (VT):** Sum of the regional relative impedance changes of the entire state image or within the defined region of interest (ROI). It is related to the increase in resistance of the lung tissue to the passage of current, which increases with the entry of air and decreases with the exit of this<br>**Minute variation (MV):** Global or regional minute tidal variation: average of the sum of the regional relative impedance changes in the last minute of the entire state image or within the defined region of interest.
20. Key secondary outcome(s)	**IPAQ:** Instrument that provides information on estimated energy expenditure in 24 hours in different areas of daily life.<br>**Charlson Index:** Relates long-term mortality to participant comorbidity<br>**%FEV1 Pred:** Percentage of predicted forced expiratory volume in the first second<br>**%FVC Pred:** Percentage of predicted forced vital capacity<br>**%FVC/FEV1:** Percentage of the predicted relationship between forced vital capacity and FEV1
